# Antidepressant-like effects of a water-soluble extract from the culture medium of *Ganoderma lucidum* mycelia in rats

**DOI:** 10.1186/1472-6882-13-370

**Published:** 2013-12-26

**Authors:** Hirokazu Matsuzaki, Yuta Shimizu, Naohiro Iwata, Shinya Kamiuchi, Fumiko Suzuki, Hiroshi Iizuka, Yasuhide Hibino, Mari Okazaki

**Affiliations:** 1 Laboratory of Pharmacology, Faculty of Pharmaceutical Sciences, Josai University, 1-1 Keyakidai, Sakado, Saitama 350-0295, Japan; 2 Laboratory of Immunobiochemistry, Faculty of Pharmaceutical Sciences, Josai University, 1-1 Keyakidai, Sakado, Saitama 350-0295, Japan; 3 Noda Shokukin-kogyo Co. Ltd.; 295 Nanakodai, Noda, Chiba 278-0051, Japan

**Keywords:** Anxiety, Depression, *Ganoderma lucidum* mycelia, Locomotion, Memory, 5-HT_2A_ receptors

## Abstract

**Background:**

*Ganoderma lucidum* is a popular medicinal mushroom used for promoting health and longevity in Asian countries. Previously, we reported that a water-soluble extract from a culture medium of *Ganoderma lucidum* mycelia (MAK) exerts antioxidative and cerebroprotective effects against ischemia–reperfusion injury *in vivo*. Here, we evaluated the antidepressant and anxiolytic activities of MAK in rats.

**Methods:**

MAK (0.3 or 1 g/kg, p.o.) was administered in the experimental animals 60 min before the forced swimming, open-field, elevated plus-maze, contextual fear-conditioning, and head twitch tests. Additionally, the mechanisms involved in the antidepressant-like action of MAK were investigated by the serotonin precursor 5-hydroxy-L-tryptophan (5-HTP)- or 5-HT_2A_ agonist (±)-1-(2,5-dimethoxy-4-iodophenyl)-2-aminopropane hydrochloride (DOI)-induced head twitch responses.

**Results:**

Treatment with MAK (1 g/kg) exhibited antidepressant-like effects in the forced swimming test, attenuated freezing behavior in the contextual fear-conditioning test, and decreased the number of head twitches induced by DOI, but not with 5-HTP. No significant response was observed in locomotion or anxiety-like behavior, when the animals were evaluated in the open-field or elevated plus-maze test, respectively.

**Conclusions:**

These data suggest that MAK has antidepressant-like potential, which is most likely due to the antagonism of 5-HT_2A_ receptors, and possesses anxiolytic-like effects toward memory-dependent and/or stress-induced anxiety in rats.

## Background

Major depressive disorder is a common, recurrent mental disease characterized by a negative mood and loss of interest or pleasure in the normal activities of daily life. It is often accompanied by a broad range of symptoms, including a decline in cognitive function, sleep disturbance and recurrent suicidality, which affects the quality-of-life (QoL) and mortality of patients. Experimental and clinical studies have suggested that major depression may be caused at least in part by the metabolic dysregulation of monoaminergic (particularly serotonin and noradrenaline) systems in the brain [1]. Drugs that increase the levels of monoamines, such as tricyclic antidepressants (TCAs), selective serotonin reuptake inhibitors (SSRIs), and serotonin and noradrenaline reuptake inhibitors (SNRIs), are the mainstays of depression therapy. However, these conventional, commercially available antidepressants provide only partial remission and frequently produce adverse effects [2]. Therefore, not all of the neurological pathogeneses of depression can be explained by the metabolic dysfunction of monoamines. As a result of these limitations, there is an appreciable unmet medical need for rapid, safe, and more effective antidepressants.

Recently, plant extracts have drawn the attention of researchers because of the importance in discovering novel antidepressant agents. Several herbal medicines (e.g., *Panax notoginseng*[3], *Asparagus racemosus*[4], *Rosmarinus officinalis*[5], and St. John’s wort [6]) have been reported to have antidepressant activity comparable with prescription medicines but with fewer side effects.

*Ganoderma lucidum* (*G. lucidum*) is a popular medicinal fungus known as Lingzhi mushroom in China. It has long been known for its beneficial effects on human health and longevity in Asian countries. *G. lucidum* has been shown to have several pharmacological effects (e.g., antitumor, immunomodulatory, anti-inflammatory, antidiabetic, antioxidative), which are supported by studies on various bioactive compounds isolated from the fruiting bodies and mycelia of this fungus [7]. A water-soluble extract prepared from the culture medium of *G. lucidum* mycelia (MAK) has a 17-year history of making appreciable contributions to consumers’ health as a safe, functional food. The extract contains various types of constituents, such as polysaccharides, including β-glucans, triterpenes, and lignin derived from the culture medium and its digestion products by the mycelia. MAK has been reported to have antitumor [8] and radioprotective effects [9].

Previously, we demonstrated that MAK has antioxidant activities and neuroprotective effects *in vivo*. Orally administered MAK can prevent ischemia–reperfusion-induced oxidative damage to neuronal cells, and reduce the size of cerebral infarcts in animal models [10,11]. However, until now, the antidepressant-like effects of MAK have not been assessed.

Therefore, the study aimed to assess the antidepressant-like and anxiolytic-like activities of MAK in rats. We performed the forced swimming test together with open-field test to evaluate antidepressant-like activity, and the elevated plus-maze test and contextual fear-conditioning test to evaluate anxiolytic-like activity of MAK. Furthermore, to ascertain if the antidepressant-like effect of MAK is mediated by the serotonergic system, we examined the effect of MAK on 5-hydroxy-L-tryptophan (5-HTP)- or (±)-1-(2,5-dimethoxy-4-iodophenyl)-2-aminopropane hydrochloride (DOI)-induced head-twitch responses.

## Methods

All experiments were conducted in accordance with the guidelines set by the National Institutes of Health (Bethesda, MD, USA) and approved by the Animal Investigation Committee of Josai University (number H23005). All possible efforts were made to minimize the number of animals utilized and their suffering.

### Animals

Male Sprague–Dawley rats (250–300 g) were purchased from Sankyo Labo Service Co. Inc. (Tokyo, Japan). Three or four animals were housed per cage in a temperature-controlled environment (23 ± 0.5°C) with a 12-h light–dark cycle (light on at 7 am) with food and water *ad libitum*. One hundred and thirty-three rats (forced swimming test, 25; open-field test, 21; elevated plus-maze test, 28; contextual fear-conditioning test, 23; 5-HTP-induced head-twitches, 18; DOI-induced head-twitches, 18) were used in the present study.

### Drug treatment

MAK was provided by Noda Shokukin-kogyo Co. Ltd. (Noda, Japan). The preparation of MAK (overall yield, ≈ 10%) was as follows: a pure culture of *G. lucidum* mycelia was inoculated into a solid culture medium that was composed of bagasse and defatted rice bran and cultured until just before the formation of the fruit body (for 3–4 months); subsequently, the entire medium overgrown with *G. lucidum* mycelia was extracted with hot water, and then the extract was sterilized by filtration and lyophilized for powderization. Fluvoxamine maleate, imipramine hydrochloride, 5-hydroxy-L-tryptophan (5-HTP), and (±)-1-(2,5-dimethoxy-4-iodophenyl)-2-aminopropane hydrochloride (DOI) were purchased from Sigma–Aldrich (St Louis, MO, USA). Drugs were dissolved in distilled water except 5-HTP and DOI, which were dissolved in saline. MAK (0.3 or 1 g/kg), imipramine (10 mg/kg) as a positive control, or distilled water was administered orally 60 min before the forced swimming test, open-field test, elevated plus-maze test, or contextual fear-conditioning test.

### Forced swimming test

The experiment was carried out according to the method described by Porsolt et al. [12] with a minor modification. Briefly, on the first day (pre-test session), rats were placed individually in a clear cylinder (20-cm diameter, 50-cm height) that contained water (25 ± 1°C) to a depth of 25 cm and forced to swim for 15 min. Subsequently, rats were removed from the cylinder, dried with towels, and warmed by a heater before being returned to their home cages. The water in the tank was replaced after it was used by each animal. On the following day (test session), rats were placed back into the cylinder for 5 min. A 5-s sampling procedure [13] was utilized for scoring the behaviors (i.e., immobility, climbing, and swimming). Immobility was noted if the rat remained floating in the water without struggling and only made movements to keep its head or nose above the water. “Climbing” was defined as the animal making upwardly directed vigorous movements of the forepaws usually along the cylinder wall while keeping its head and shoulders above the water. “Swimming” was defined as horizontal movement throughout the cylinder with a more vigorous motion of all four paws than that necessary to merely maintain the head above the water.

### Open-field test

Locomotor activity was assessed by the open-field test following a protocol described previously [14]. The open-field apparatus comprised a gray box (70 cm × 70 cm; 40-cm height) divided into 49 squares of equal area (10 cm × 10 cm). At the start of each test, rats were placed in the center of the open field and allowed to explore the inside of the box. Behavior was monitored using a video camera that was positioned above the apparatus during the 30-min test period. The number of lines crossing was analyzed automatically by a video-tracking system with a software package (CompACT VAS, Muromachi Kikai, Tokyo, Japan). The apparatus was cleaned with isopropanol after each test.

### Elevated plus-maze test

To examine the exploratory behavior of rats, the elevated plus-maze was performed as described previously [15] with minor modifications. The apparatus (which was raised 50 cm above the floor) consisted of two opposing open arms (50-cm long × 10-cm wide) and two opposing closed arms of the same size surrounded by side walls (40-cm high) that extended from the central platform (10 cm × 10 cm). At the start of each test, rats were placed individually on the central platform, and their behaviors monitored by the video camera for 10 min. The number of entries for each arm and the time spent in each arm were recorded and analyzed automatically using CompACT VAS. The number of closed arm entries was used as an index of locomotor activity. The percentage of open arm entries (open arm entries × 100/ total arm entries) and percentage of time spent in open arm (time spent in open arm × 100/time spent in open and closed arms) were used as indices of anxiety.

### Contextual fear-conditioning test

A footshock chamber that consisted of a plexiglass rectangular box (30 cm × 30 cm × 30 cm) with a floor of 18 stainless-steel rods (6-mm diameter) wired to a shock generator (LE 100-26, Panlab, Barcelona, Spain) was used to evaluate memory-dependent fear-related behavior. The experiment was done during 2 consecutive days. On the first day, each rat was exposed to the shock chamber for 5 min for adaptation to the experimental conditions, and then the rat received five inescapable electrical footshocks for a total of 2.5 min (0.5 mA, 2-s duration, repeated every 30 s). Five minutes after the last footshock, the rat was returned to his home cage. The following day (24 h later), the rat was placed into the same footshock chamber without footshocks. In the 10-min observation period, freezing behavior was recorded using a time-sampling procedure [16] in which animal behavior was classified as “freezing” or “activity” at 5-s intervals. Freezing behavior was defined as the absence of all movement with the exception of movements related to respiration and whisker movements [16]. The percentage scores for freezing were calculated for a 10-min observation period. Analyses of freezing behavior were evaluated by an investigator blinded to the treatment protocol.

### Head-twitch test

The head-twitch test was carried out according a method described by Rojas-Corrales et al. [17] with minor modifications. Rats were orally treated with MAK (1 g/kg) or fluvoxamine (10 mg/kg) 60 min before intraperitoneal injection of 5-HTP (200 mg/kg). Immediately after 5-HTP administration, the rat was placed into an observation cage (30 cm × 25 cm × 18 cm) and the cumulative number of head twitches (i.e., rapid movements of the head with little or no involvement of the trunk) counted by an observer blinded to the study protocol for 30 min. Similarly, the effect of MAK on DOI (3 mg/kg)-induced head-twitch responses was measured.

### Statistics analyses

Data are the mean ± S.E.M. Significant differences between groups were evaluated using one-way analysis of variance (ANOVA), followed by the Tukey’s *post hoc* test except for contextual fear-conditioning test, which by the Bonferroni’s test. P < 0.05 was considered significant. The statistical analysis was conducted using GraphPad Prism software (Graphpad Software, Inc., San Diego, CA, USA).

## Results

### Forced swimming test

**Figure 1:**
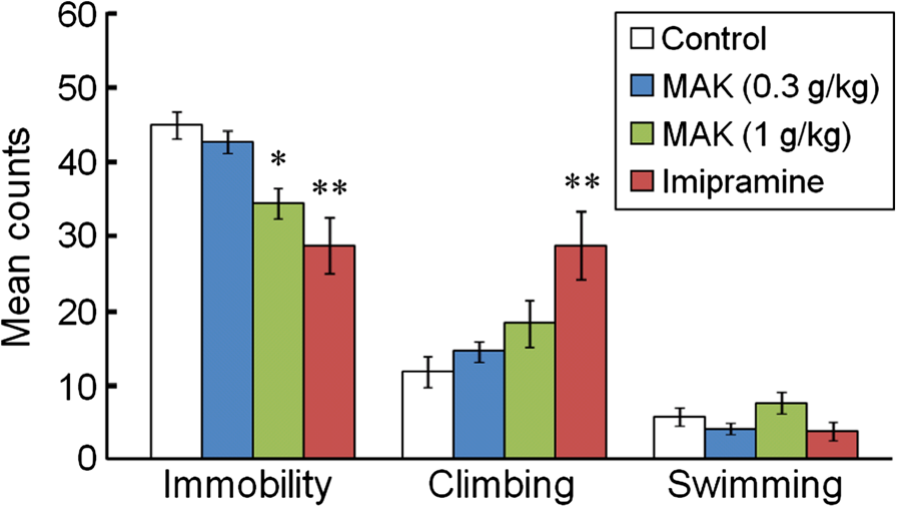
**Effects of MAK on the duration of immobility, climbing and swimming behaviors in the forced swimming test.** Behaviors were scored every 5 s for a 5-min observation period. Results are the mean ± S.E.M. The number of rats per group was: control group, n = 8; MAK (0.3 g/kg)-treated group, n = 6; MAK (1 g/kg)-treated group, n = 6; imipramine-treated group, n = 5. *P < 0.05, **P < 0.01 compared with the control group, one-way analysis of variance followed by Tukey’s test.

Figure 1 illustrates the effects of MAK on immobility, climbing, and swimming behaviors in the forced swimming test. MAK (1 g/kg) and the classical antidepressant imipramine reduced immobility (23% and 35%, respectively) in the forced swimming test compared with the distilled water-treated control group. One-way ANOVA revealed significant differences in immobility ([F(3,21) = 11.09, P < 0.01]). *Post hoc* analyses indicated significant differences between the control group and the MAK (1 g/kg)-treated group (P < 0.05) and the imipramine-treated group (P < 0.01). Furthermore, one-way ANOVA revealed a significant differences in climbing ([F(3,21) = 7.33, P < 0.01]). *Post hoc* analyses indicated a significant difference between the control group and the imipramine-treated group (P < 0.01), but the difference between the control group and MAK-treated group was not significant.

### Open-field test

**Figure 2:**
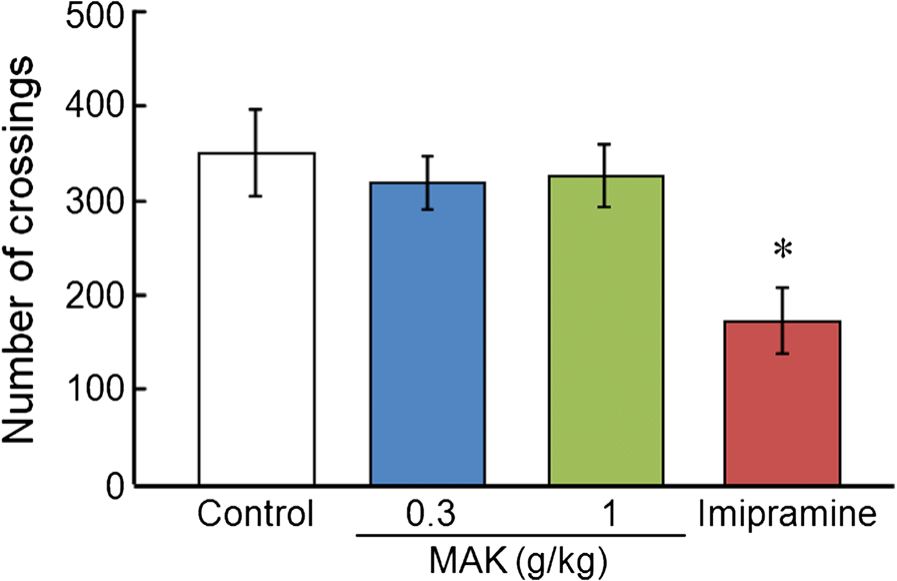
**Effects of MAK on locomotor activity in the open-field test.** Results are the mean ± S.E.M. The number of crossings recorded for a 30-min period. The number of rats per group was: control group, n = 6; MAK (0.3 g/kg)-treated group, n = 5; MAK (1 g/kg)-treated group, n = 5; imipramine-treated group, n = 5. *P < 0.05 compared with the control group, one-way analysis of variance followed by Tukey’s test.

To exclude the possibility of a false-positive effect in the forced swimming test, the effects of MAK on locomotor activity were tested. One-way ANOVA revealed significant differences in locomotor activity ([F(3,17) = 3.55, P < 0.05]). *Post hoc* analyses indicated a significant difference between the control group and the imipramine-treated group (P < 0.05) (Figure 2).

### Elevated plus-maze test

No significant differences were detected for rats treated with MAK or imipramine compared with control rats on the number of closed arm entries ([F(3,24) = 0.79, P = 0.51]), the percentage of open arm entries ([F(3,24) = 0.08, P = 0.97]), or the percentage of time spent in open arms ([F(3,24) = 0.09, P = 0.97]) (Table 1).

**Table 1:** Effects of MAK on the elevated plus-maze test

	**Number of closed arm entries**	**Percentage of open arm entries**	**Percentage of time spent in open arm**
Control	18.0 ± 4.4	50.2 ± 4.0	36.2 ± 7.8
MAK (0.3 g/kg)	23.0 ± 4.2	47.8 ± 5.8	34.5 ± 11.0
MAK (1 g/kg)	15.0 ± 3.5	47.8 ± 6.1	42.0 ± 13.9
Imipramine	18.8 ± 4.1	46.1 ± 6.0	41.1 ± 13.7

Results are the mean ± S.E.M. The number of rats per group was: control group, n = 7; MAK (0.3 g/kg)-treated group, n = 6; MAK (1 g/kg)-treated group, n = 9; imipramine-treated group, n = 6.

### Contextual fear-conditioning test

**Figure 3:**
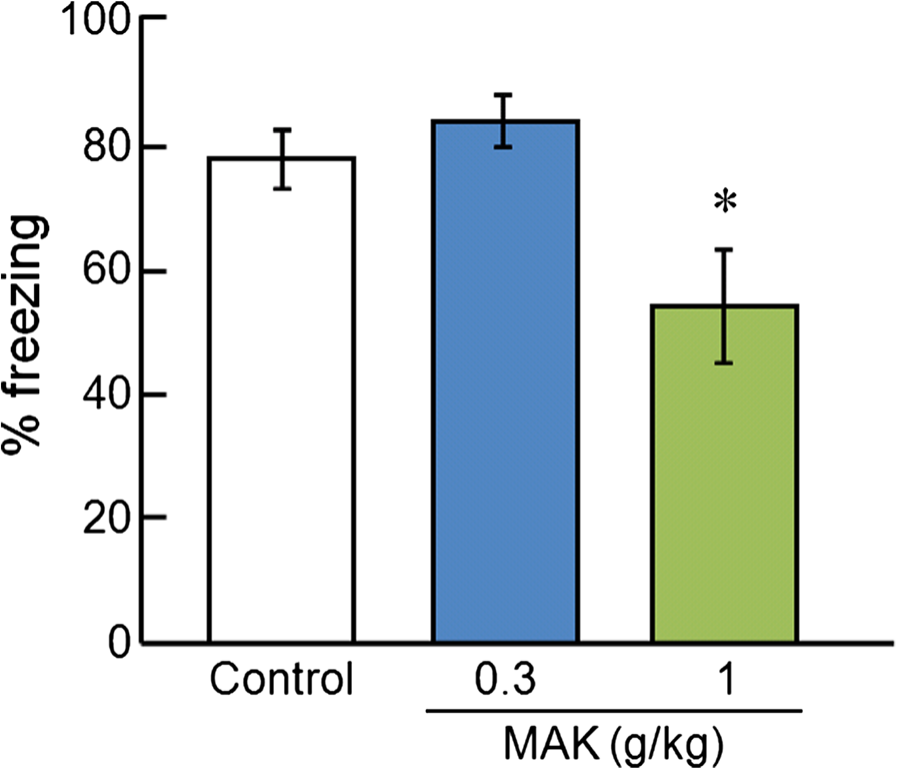
**Effects of MAK on freezing behavior in the contextual fear-conditioning test.** Results are the mean ± S.E.M. of freezing scored for a 10-min observation period. The number of rats per group was: control group, n = 9; MAK(0.3 g/kg)-treated group, n = 6; MAK (1 g/kg)-treated group, n = 8. *P < 0.05 compared with the control group, one-way analysis of variance followed by Bonferroni’s test.

In the contextual fear-conditioning test, the freezing rate was decreased to 49.6 ± 8.5% in the MAK (1 g/kg)-treated group compared with 72.8 ± 3.5% in the control group (Figure 3). One-way ANOVA revealed significant differences in freezing behavior ([F(2,20) = 8.39, P < 0.01]). *Post hoc* analyses indicated a significant difference between the control group and the MAK (1 g/kg)-treated group (P < 0.05).

### 5-HTP- or DOI-induced head twitches

**Figure 4:**
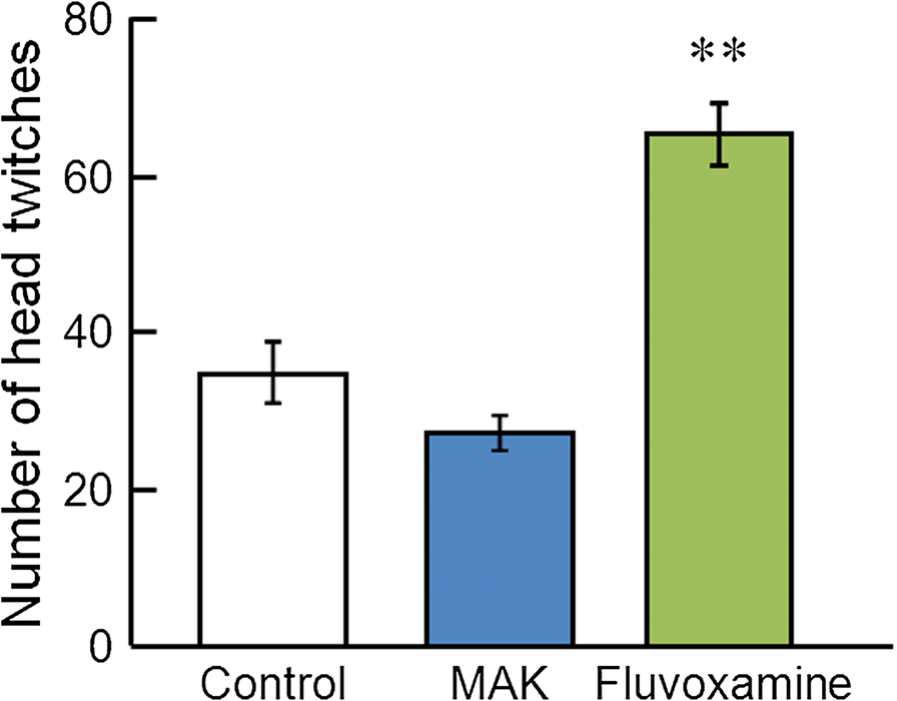
**Effects of MAK on 5-HTP-induced head twitches.** Results are the mean ± S.E.M. of the number of head twitches for a 30-min observation period. The number of rats per group was: control group, n = 6; MAK-treated group, n = 6; fluvoxamine-treated group, n = 6. **P < 0.01 compared with the control group, one-way analysis of variance followed by Tukey’s test.

**Figure 5:**
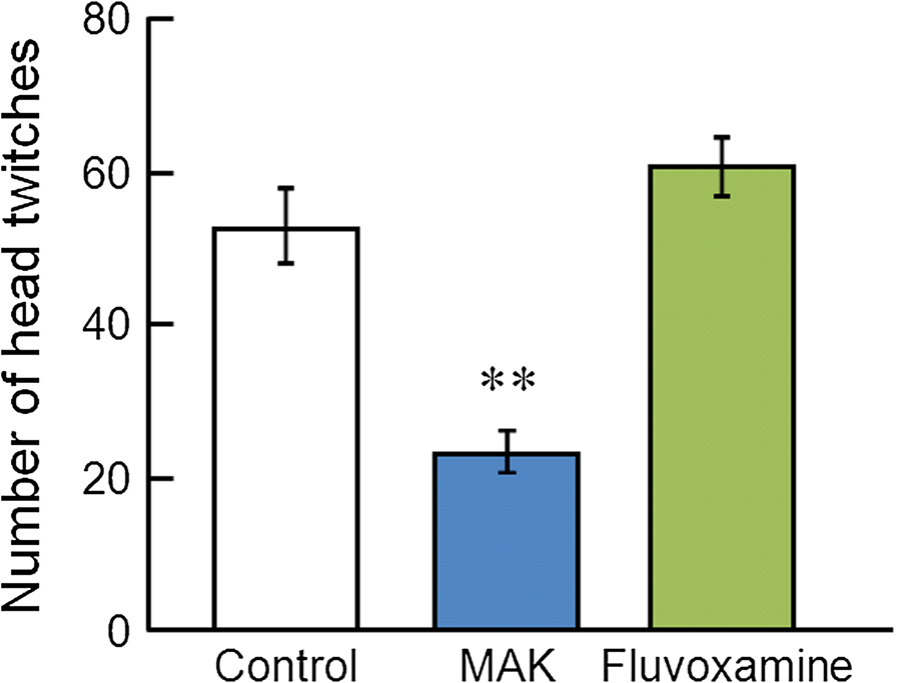
**Effects of MAK on DOI-induced head twitches.** Results are the mean ± S.E.M. of the number of head twitches for a 30-min observation period. The number of rats per group was: control group, n = 6; MAK-treated group, n = 6; fluvoxamine-treated group, n = 6. **P < 0.01 compared with the control group, one-way analysis of variance followed by Tukey’s test.

Figure 4 shows the effect of MAK (1 g/kg) on 5-HTP-induced head twitches. One-way ANOVA revealed significant differences in 5-HTP-induced head twitches ([F(2,15) = 33.4, P < 0.01]). *Post hoc* analyses indicated significant differences between the control group and fluvoxamine-treated group (P < 0.01), but the difference between the control group and MAK-treated group was not significant (P = 0.33). Figure 5 demonstrates the effect of MAK on DOI-induced head twitches. One-way ANOVA revealed significant differences in DOI-induced head twitches ([F(2,15) = 25.2, P < 0.01]). *Post hoc* analyses indicated significant differences between the control group and MAK-treated group (P < 0.01).

## Discussion

In the present study, we provided evidence, for the first time, that acute oral administration of MAK (a functional food derived from *G. lucidum* mycelia) exerts a significant antidepressant-like effect in rats, and that this effect can be observed in the forced swimming test. The forced swimming test is the most widely used paradigm for screening potential antidepressants in rodents. A significant correlation between the efficacy and effectiveness of therapy has been demonstrated in the forced swimming test [12]. Various antidepressants, such as TCAs, SSRIs, and SNRIs, have been demonstrated to reduce immobility without altering locomotor activity [18]. The present study showed that MAK significantly reduced immobility in the forced swimming test. Psychostimulants, such as amphetamine and caffeine, also reduce immobility in the forced swimming test with increasing general activity [12], so MAK was evaluated for its effects on locomotor activity in the open-field test to exclude a false-positive effect. At a dose that showed a significant decrease in immobility in the forced swimming test (1 g/kg), MAK did not change locomotor activity. These results suggest that the MAK-induced decrease in immobility in the forced swimming test is caused by an antidepressant-like effect rather than a locomotor-enhancing effect.

To evaluate the anxiety-like effect of MAK, the contextual fear-conditioning test and elevated plus-maze test as memory-dependent and -independent tasks, respectively, were undertaken [19,20]. In the contextual fear-conditioning test, the MAK (1 g/kg)-treated group showed a significant decrease in freezing behavior compared with the control group. By contrast, MAK did not affect the index of anxiety (% open arm entries and % time spent in open arm) or locomotor activity (closed arm entries) in the elevated plus-maze test. Conditioned fear-induced freezing behavior has been demonstrated to be decreased by representative anxiolytic agents such as benzodiazepines [21] and SSRIs [22]. Similarly, the percentage of time spent in the open arms in the elevated plus-maze test is increased by treatment with clinically effective anxiolytic agents but is decreased by anxiogenic agents [23]. However, the elevated plus-maze test is known to exhibit remarkable variability, and sometimes paradoxical patterns in experimental results using 5-HT-related agents have been obtained [24,25]. Indeed, the elevated plus-maze test could not detect the anxiolytic-like effects of 5-HT_2_ receptor antagonists such as ketanserin, mianserin, and MDL-100907 in previous studies [26,27]. Collectively, the elevated plus-maze test might not have been suitable to assess the anxiolytic effect of MAK, which may possess anxiolytic-like effects toward memory-dependent and/or stress-induced anxiety.

The head-twitch response is defined as a rapid movement of the head and neck. It is a useful index for assessing the effects of drugs on central serotonergic activity *in vivo* in rodent experiments. The stereotypical behavior is induced by the serotonin precursor 5-HTP via an increase of serotonin levels in the synaptic clefts [28] and the subsequent indirect activation of 5-HT_2A_ receptors [29]. This response has been reported to be enhanced by acute inhibition of 5-HT reuptake by SSRIs [30]. By contrast, DOI, which has an approximately equal affinity for 5-HT_2A_, 5-HT_2B_ and 5-HT_2C_ receptors, is considered to induce a head-twitch response by a direct agonistic effect on 5-HT_2A_ receptors, because a DOI-induced head-twitch response is blocked by selective 5-HT_2A_ antagonists but not by 5-HT_2B/2C_ antagonists [29,31] and is not modified by SSRIs [17,31]. The present study confirmed that the oral administration of the SSRI fluvoxamine (10 mg/kg), which inhibits the 5-HT reuptake and increases extracellular 5-HT concentration in the brain [32], augments the head twitches induced by 5-HTP but does not change those induced by DOI. In contrast, MAK significantly decreased the number of head twitches induced by DOI without altering that induced by 5-HTP. These results suggest that MAK exerts a 5-HT_2A_ receptor inhibitory effect rather than a 5-HT reuptake-inhibitory effect. The results for MAK are similar to those from a previous study that indicated that the antidepressant nefazodone (which is a potent 5-HT_2A_ antagonist with a low affinity for serotonin transporters) significantly attenuated the head twitches induced by DOI but did not affect those induced by 5-HTP [17]. Previous studies have shown that the 5-HT_1A_ agonist 8-OH-DPAT also attenuates the DOI-induced head twitch response in rats and mice [31]. However, it is unlikely that the effect of MAK involves the 5-HT_1A_ mechanisms, because we observed no serotonin syndrome-like behaviors (such as flat body posture, hind limb abduction, and forepaw treading) that are induced primarily by an agonism at 5-HT_1_ receptor subtypes [31] in the MAK-treated rats. Studies on the action of *G. lucidum* on serotonin receptors are lacking, but a recent study focusing on the antipruritic effect of a methanol extract of the fruiting body of *G. lucidum* showed that the extract inhibits the scratching behavior induced by intradermal administration of serotonin or a 5-HT_2_ receptor agonist alpha-methyl-5-HT in mice [33]. Taken together, the findings of the present study suggest that the antidepressant-like effect of MAK might be mediated (at least in part) by 5-HT_2A_ receptor inhibition in the brain. On the other hand, the antidepressant mechanism of MAK may also involve the noradrenergic system in addition to the serotonergic system. Future studies will also address the role of the noradrenergic system in the mechanism of action of MAK.

Studies in humans and in animal models of depression have provided evidence that oxidative stress and subsequent inflammatory neurodegenerative responses have an important pathophysiological role in depression [34]. Neuronal cells are known to be especially prone to oxidative damage because of their higher oxygen consumption and inadequate antioxidant defense systems to reactive oxygen species [35]. Several studies have demonstrated lowered antioxidant defenses (such as decreases in the levels of antioxidants and antioxidant enzymes) and increased oxidative stress (such as increased levels of lipid peroxidation and DNA damage) in patients with major depression [34]. Moreover, antidepressant drugs have been demonstrated to ameliorate oxidative disturbances in depressed patients [36] and chronically stressed animals [37]. Previously, we reported that MAK exerts radical-scavenging activity and suppresses lipid peroxidation in a concentration-dependent manner *in vitro*[38]. Additionally, oral administration of MAK (1 g/kg/day) for 2 weeks has been shown to normalize the augmentation of oxidative stress and antioxidant enzyme (superoxide dismutase, catalase, and glutathione peroxidase) activities in streptozotocin-induced diabetic rat brains [10]. Furthermore, we demonstrated that MAK could prevent ischemia–reperfusion-induced oxidative damage and subsequent inflammatory responses in neuronal cells, and reduce the size of cerebral infarcts in animal models [10,11]. Therefore, the antioxidant and neuroprotective activities of MAK could contribute (at least in part) to its antidepressant-like effects. However, it is uncertain how such anti-oxidative effects brought about by acute administration of MAK affect the behaviors in the tests we observed in this report.

MAK has a 17-year history of making appreciable contributions to consumers’ health as a safe, functional food. In the past, there was no report suggesting that MAK had potential acute or long-term toxicities in consumers. The safety of MAK has been confirmed by *in vitro* toxicological evaluation and animal toxicity studies. Additionally, we showed previously that chronic treatment of MAK (1 g/kg for 9 weeks) had no effect on body-weight gain, levels of aspartate aminotransferase, alanine aminotransferase, triglycerides and total cholesterol in the serum, or the histochemistry in the brains, livers and kidneys of healthy mice [38]. Therefore, single administration of 1 g/kg MAK used in the present study is considered to have little or no toxicity. MAK is composed of bagasse and defatted rice bran that is overgrown with *G. lucidum* mycelia, which is considered to contain various bioactive substances, including triterpenes, polysaccharides and water-soluble lignin. Recently, triterpenes from *Bacopa monnieri*[39] and *Centella asiatica*[40] as well as polysaccharides from *Panax ginseng*[41] were shown to have antidepressant-like effects in animals. Accordingly, MAK may also contain active constituents that exert similar pharmacological effects. Future works need to be conducted to identify the substances that contribute to the antidepressant effects of MAK.

## Conclusion

In summary, the present study demonstrated that MAK has antidepressant-like potential, which is most likely as a result of the antagonism of 5-HT_2A_ receptors, and possesses anxiolytic-like effects toward memory-dependent and/or stress-induced anxiety in rats. Hereafter, further chemical and pharmacological analyses of MAK will be conducted to isolate and characterize the active ingredients responsible for these antidepressant-like effects.

## Abbreviations

5-HT: 5-hydroxytryptamine (serotonin); 5-HTP: 5-hydroxytryptophan; DOI: (±)-1-(2,5-dimethoxy-4-iodophenyl)-2-aminopropane hydrochloride; MAK: A water-soluble extract from the culture medium of *Ganoderma lucidum* mycelia; SSRIs: Selective serotonin reuptake inhibitors; SNRIs: Serotonin and noradrenaline reuptake inhibitors; TCAs: Tricyclic antidepressants.

## Competing interests

The authors declare that they have no competing interests.

## Authors’ contributions

MO and YH conceived the project and supervised the study. HM designed the experimental protocol, carried out the behavioral tests with YS, analyzed the data, and wrote the first draft of the manuscript. NI and SK helped to develop the protocol and analyze the data. FS and HI prepared a water-soluble extract from the culture medium of *Ganoderma lucidum* mycelia. MO evaluated the results and wrote the final draft of this manuscript. All of the authors have approved the final manuscript.

## Pre-publication history

The pre-publication history for this paper can be accessed here:

http://www.biomedcentral.com/1472-6882/13/370/prepub
